# Household transmission of Omicron variant of SARS-CoV-2 under conditions of hybrid immunity—a prospective study in Germany

**DOI:** 10.1007/s15010-024-02352-4

**Published:** 2024-07-22

**Authors:** Bianca Klee, Sophie Diexer, Chao Xu, Cornelia Gottschick, Carla Hartmann, Kristin Maria Meyer-Schlinkmann, Alexander Kuhlmann, Jonas Rosendahl, Mascha Binder, Michael Gekle, Matthias Girndt, Jessica I. Höll, Irene Moor, Daniel Sedding, Stefan Moritz, Thomas Frese, Rafael Mikolajczyk

**Affiliations:** 1 Institute for Medical Epidemiology, Biometrics and Informatics (IMEBI), Interdisciplinary Centre for Health Sciences, Medical Faculty of the Martin Luther University Halle-Wittenberg, Magdeburger Str. 8, 06112 Halle (Saale), Germany; 2 MVZ Labor Krone eGbR, Siemensstraße 40, 32105 Bad Salzuflen, Germany; 3 Faculty of Medicine, Martin Luther University Halle-Wittenberg, Magdeburger Str. 8, 06112 Halle (Saale), Germany; 4 Department of Internal Medicine I, Martin Luther University Halle-Wittenberg, Ernst-Grube-Str. 40, 06120 Halle (Saale), Germany; 5 Department of Internal Medicine IV, Oncology/Haematology, Martin Luther University Halle-Wittenberg, Ernst-Grube-Str. 40, 06120 Halle (Saale), Germany; 6 Medical Oncology and Laboratory for Translational Immuno-Oncology, Universitätsspital Basel, Basel, Switzerland; 7 Julius-Bernstein-Institute of Physiology, Medical Faculty of the Martin Luther University Halle-Wittenberg, Magdeburger Str. 6, 06110 Halle (Saale), Germany; 8 Department of Internal Medicine II, Martin Luther University Halle-Wittenberg, Ernst-Grube-Str. 40, 06120 Halle (Saale), Germany; 9 Paediatric Haematology and Oncology, Martin Luther University Halle-Wittenberg, Ernst-Grube-Str. 40, 06120 Halle (Saale), Germany; 10 Institute of Medical Sociology, Martin Luther University Halle-Wittenberg, Magdeburger Str. 8, 06112 Halle (Saale), Germany; 11 Mid-German Heart Centre, Department of Cardiology and Intensive Care Medicine, University Hospital, Martin Luther University Halle-Wittenberg, Ernst-Grube-Str. 40, 06120 Halle (Saale), Germany; 12 Section of Clinical Infectious Diseases, University Hospital Halle (Saale), Martin Luther University Halle-Wittenberg, Ernst-Grube-Str. 40, 06120 Halle (Saale), Germany; 13 Institute of General Practice and Family Medicine, Interdisciplinary Centre for Health Sciences, Medical Faculty of the Martin Luther University Halle-Wittenberg, Magdeburger Str. 8, 06112 Halle (Saale), Germany

**Keywords:** Household transmission, Protection, Omicron variant, Waning immunity, Digital cohort

## Abstract

**Purpose:**

We investigated the protection offered by vaccinations and previous infections for the household transmission of Omicron variant of SARS-CoV-2.

**Methods:**

34,666 participants of the German DigiHero cohort study with two or more household members were invited to a prospective household transmission study between June and December 2022. In case of a positive SARS-CoV-2 test in a household, symptom diaries were completed for at least 14 days. Dry blood spots (DBS) were taken from all household members at the beginning and six to eight weeks later. DBS were analyzed for SARS-CoV-2 antibodies.

**Results:**

1191 individuals from 457 households participated. The risk of acquiring a SARS-CoV-2 infection decreased with higher S-titer levels at the time of exposure (from 80% at titer of 0 binding antibody units (BAU)/ml to 20% at titer of 3000 BAU/ml) and increased linearly with the time since vaccination/previous infection (20% for less than one month to 80% at one year). Transmission probability was also reduced when the symptoms of the primary case were mild and if preventive measures were implemented.

**Conclusion:**

Vaccinations/previous infections offer a high protection against infection with the Omicron variant for a few months only, supporting the notion of seasonal circulation of the virus.

**Supplementary Information:**

The online version contains supplementary material available at 10.1007/s15010-024-02352-4.

## Introduction

In May 2023, the WHO declared that the COVID-19 pandemic was no longer a global health emergency [1]. SARS-CoV-2 became an endemic pathogen, and since the end of 2021, the Omicron variant dominates in many countries [2]. The Omicron variant has a higher transmissibility and has a high ability to escape immunity, while it causes more mild diseases and less hospitalizations compared to the previous variants [3]. The high transmissibility was shown in a meta-analysis in early 2022, where Madewell et al. estimated the highest secondary attack rate (SAR) for Omicron with 42.7%, followed by Alpha (36.4%) and Delta (29.7%) [4]. These estimates partly included vaccinated individuals, making a direct comparison difficult. From other research, it is known that vaccinations less effectively reduced transmission for Omicron compared to Alpha and Delta variants [5–7]. Similarly, a recent meta-analysis found that the protection offered by a previous infection was lower for Omicron and declined more rapidly over time compared to previous variants [8].

Besides vaccinations, less symptoms or even asymptomatic course of infection are associated with a lower risk of transmission. Furthermore, preventive measures like isolation of infected persons, mask wearing at home, disinfection, were shown to reduce the transmission of SARS-CoV-2 in households [9–12]. While several studies have already investigated the waning of protection offered by vaccination or previous infection with SARS-CoV-2, most of these studies applied a test negative design or retrospective methodology [8, 13]. There are only a few studies investigating protective antibody titers with real world data [14, 15], and they were neither done in household settings, which allows to define the timing of exposure, nor during the Omicron pandemic.

Therefore, this study aimed to determine the risk of acquiring infection from household exposure to the Omicron variant in a community setting with mixed immunity. Particularly, we wanted to estimate the effect of titer decrease and the time since last exposure (vaccination or infection) on the risk of acquiring an infection in the household.

## Methods

### Study design and participants

This study was conducted within the prospective cohort study for digital health research in Germany (DigiHero, DRKS Registration-ID: DRKS00025600). DigiHero is a nationwide study with currently over 90,000 participants (11/2023) and an ongoing recruitment. The study was initiated in 2021, with the overall goal to establish a digital research platform for rapid collection of health related data. DigiHero is population-based, with postal invitations sent to a random sample of persons aged 18–87 from the local registry offices in selected federal states in Germany as previously described [16]. Those interested register online and fill out a baseline questionnaire. Further questionnaires and invitations to other modules are sent out electronically three to four times per year.

### Module on transmission in households

In June 2022, we initiated a substudy on the transmission of SARS-CoV-2 in the household setting among DigiHero participants, who have been recruited at this time. In total, 34,666 participants from households with two or more household members were invited. Those who expressed interest in participating in the substudy (n = 11,492) were asked to send an email to our study team within 24 h if a member of the household has been tested positive for SARS-CoV-2. Monthly reminders were sent to those with interest in participating. We defined a positive test as a positive PCR test result, one positive rapid test with symptoms, or two or more positive rapid tests without symptoms. The study team prepared a parcel with all study materials and sent it to the participants via postal mail. The parcel usually arrived the day after the study center was notified of the positive SARS-CoV-2 test. We ended the study in December 2022.

We asked the participants to collect dry blood spots from all household members using an established kit (see below) as soon as the parcel arrived at their home. All participants completed a symptom diary for the following 14 days whereby parents could fill in symptom diaries for their children. Symptoms included fever, cough, sore throat, runny nose, headache, and limb pain, tiredness, mostly lying in bed, diarrhea, vomiting, nausea, disturbance of smell or taste, insomnia, shortness of breath, and other symptoms. Participants were asked to report the intake of medications, medical consultations, stays in hospital, and tests for SARS-CoV-2. After all participants in the household recovered from their infection, we asked one person to complete an online survey about the timing and course of infections, the measures used to avoid transmission (disinfection, separate meal times, staying in separate rooms, wearing masks, keep distance, others), and previous infections and vaccinations of all household members. We sent dry blood spot kits again approximately six to eight weeks after the initial infection in the household.

The Ethics Committees of the Martin Luther University Halle-Wittenberg, Germany (No. 2020-076) approved the study. Participants received detailed information on the objective and the procedure of the study and provided written informed consent.

### Laboratory analyses

Participants collected a capillary blood sample via self-sampling. For this purpose, we used a Dry Blood Spot (DBS) card from AHLSTROM-MUNKSJÖ [17]. Participants were instructed to drop the capillary blood directly from the fingertip onto the DBS cards and let it dry for at least two hours. DBS cards were stored at room temperature before analysis. Two spots with a diameter of 4.7 mm were punched per card. We discarded the DBS if it was not possible to get two soaked blood spots. DBS were eluted by addition of 500 µl Sample Buffer (Euroimmun) in deep-well plates (1 ml, Euroimmun), shaked for 30 s at 700–1000 rpm and incubated for 1 h at 37 °C. All DBS eluates were centrifuged by room temperature for 5 min and 5,000 rpm. DBS holders (Euroimmun) were put in the wells in order to fix the spots on the bottom of the well. Analysis for SARS-CoV-2 antibodies (EUROIMMUN Anti-SARS-CoV-2- QuantiVac-ELISA (IgG) & EUROIMMUN-Anti-SARS-CoV-2-NCP-ELISA (IgG)) was performed using ELISA (EUROLabWorkstation, Euroimmun). Laboratory analysis was performed by the German lab “MVZ Labor Krone eGbR”.

### Definitions

In order to establish the occurrence of transmission, we combined information regarding positive tests and changes for anti-spike protein antibody titer (S-titer) and for anti-nucleocapsid protein antibody (N-titer) between the sample collected in the beginning and 6–8 weeks later. We used a stepwise approach. First, we classified all those who reported a positive SARS-CoV-2 test regardless of the type of test (rapid test or PCR) as new infections. After that, all participants with a seroconversion (change from negative to positive titer) of N- or S-titer were added. Finally, participants with a 1.5-fold increase for either N- or S- titer were considered as having been infected. We performed a sensitivity analysis in which individuals defined as infected due to the 1.5-fold increase in the antibody titers were classified as not infected. We defined individuals who first became infected in the household as index cases. If the infection of other household members started on the same day, they were classified as simultaneously infected, if there was a delay of 1–14 days they were classified as secondary cases. Households with simultaneous infections, in which there were no further uninfected household members were excluded.

To determine the symptoms of the index case, an adapted version of the acute respiratory illness (ARI) definition was used [18]. The index case was classified as having an ARI if the person (1) had fever or was so sick that she/he had to stay in bed for at least 1 day or (2) had two consecutive days with at least one respiratory symptom (cough, sore throat, cold, dyspnea) and one systemic symptom (headache, muscle ache, fatigue, or sleep disorder). If this definition was not met, the index case was either classified as mildly symptomatic (some respiratory or systemic symptoms but not meeting ARI threshold) or as asymptomatic (no symptoms reported).

### Statistical analysis

We report frequencies and mean values for descriptive statistics. After defining putative transmission chains, we used generalized additive regression models to assess the association between antibody levels of the S-titer at the time-point of exposure to an infectious household member or the time since last preceding exposure (vaccination or infection) and the risk of acquiring an infection [19]. In addition, we calculated the secondary attack rate (SAR) for households, dividing secondary infections by the number of all household members. Univariable and multivariable logistic regression was used to identify factors associated with the risk of acquiring an infection.

Analysis was conducted in R (version 4.3.1) and 95% confidence intervals (CIs) are reported for all analyses.

## Results

### Study population and SAR

We recruited 1191 individuals from 457 households for the study. We excluded 289 household members with missing consent and/or serology at any of the two time points. 54 households were then left with only one participant and excluded. In 48 households, we could not clearly identify the occurrence of transmission because of missing dates or simultaneous infections in two person households, resulting in a final sample size of 262 households with 662 participants for the analysis of transmission (Figure S1). There were slightly more female participants, 4 out of 5 participants were adults, and two person households were most common (Table 1). Most of the household members with previous infections were also vaccinated (78%) while the proportion of previously infected among those vaccinated was lower (46%) (Table S1).

**Table 1:** Characteristics of participants divided into primary cases and household contacts and individual secondary attack rate (SAR)

	Total	Primary cases*	Other household members**	Individual SAR in % (95% CI)
*Overall*	662	273	389	58 (53;63)
*Sex*				
Male	321 (48.5)	124 (45.4)	197 (50.6)	59 (52;66)
Female	340 (51.4)	148 (54.2)	192 (49.4)	56 (48;63)
Diverse	1 (0.2)	1 (0.4)		
*Age*				
Child	122 (18.4)	33 (12.1)	89 (22.9)	40 (30; 51)
Adult	540 (81.6)	240 (87.9)	300 (77.1)	63 (57; 68)
*Household size*				
2 persons	284 (42.9)	142 (52.0)	142 (36.5)	65 (56;73)
3 persons	176 (26.6)	72 (26.4)	104 (26.7)	53 (43;63)
4 persons	156 (23.6)	49 (17.9)	107 (27.5)	53 (43;63)
5 + persons	46 (6.9)	10 (3.7)	36 (9.3)	56 (38;72)
*Household composition*				
Adults only	369 (55.7)	178 (65.2)	191 (49.1)	65 (58;72)
1 child	162 (24.5)	60 (22.0)	102 (26.2)	47 (37;57)
2 children	116 (17.5)	32 (11.7)	84 (21.6)	56 (45;67)
3 + children	15 (2.3)	3 (1.1)	12 (3.1)	42 (15;72)
*Previous vaccinations*				
0	83 (12.5)	22 (8.1)	61 (15.7)	48 (35;61)
1–2	130 (19.6)	50 (18.3)	80 (20.6)	49 (37;60)
3	401 (60.6)	180 (65.9)	221 (56.8)	62 (55;68)
4	46 (6.9)	21 (7.7)	25 (6.4)	72 (51;88)
Missing	2 (0.3)		2 (0.5)	
*Previous infections*				
0	380 (57.4)	183 (67.0)	197 (50.6)	73 (66;79)
1	240 (36.3)	79 (28.9)	161 (41.4)	45 (37;53)
2	38 (5.7)	10 (3.7)	28 (7.2)	21 (8;41)
Missing	4 (0.6)	1 (0.4)	3 (0.8)	
*Vaccine or infection in last 3 months*				
No	585 (88.4)	250 (91.6)	335 (86.1)	63 (57;68)
Yes	77 (11.6)	23 (8.4)	54 (13.9)	26 (15;40)
*Positive S-titer or N-titer at baseline DBS*				
No	253 (38.2)	99 (36.3)	154 (39.6)	71 (65;77)
Yes	409 (61.8)	174 (63.7)	235 (60.4)	36 (29;44)

*Primary cases are the first infected persons in the household

**Other household members are all other participants beside primary cases

Of the 389 household members at risk, 224 were classified as secondary cases, yielding a SAR of 58% (95% CI 53–63%). Of them, 142 (63.3%) were classified as secondary cases based on a positive SARS-CoV-2 test, 24 (10.7%) based on seroconversion, and 58 (25.9%) on the 1.5-fold titer increase. The median interval between index patient onset date and household contact onset date was 3 days (IQR = 2–4 days).

We classified 168 households (64.1% of all, and 30.5% of households with more than two persons) as displaying transmission events. Most index cases (85.0%, 232 of 273) and household contacts (65.6%, 147 of 224) reported COVID-19–compatible symptoms. The majority of the household contacts with a positive SARS-CoV-2 had symptoms. The symptomatic proportions in the secondary cases were similar among those with seroconversion and titer increase (Table S2). The SAR was higher if the index case had COVID-19–compatible symptoms (59%, 95% CI 53–64%) compared to if the index case had mild symptoms (52%, 95% CI 39–65%) or no symptoms (40%, 95% CI 5–85%). Various measures were employed to reduce transmission in the households (Table S3).

### Risk of acquiring infection depending on preceding vaccinations or infections

**Fig. 1:**
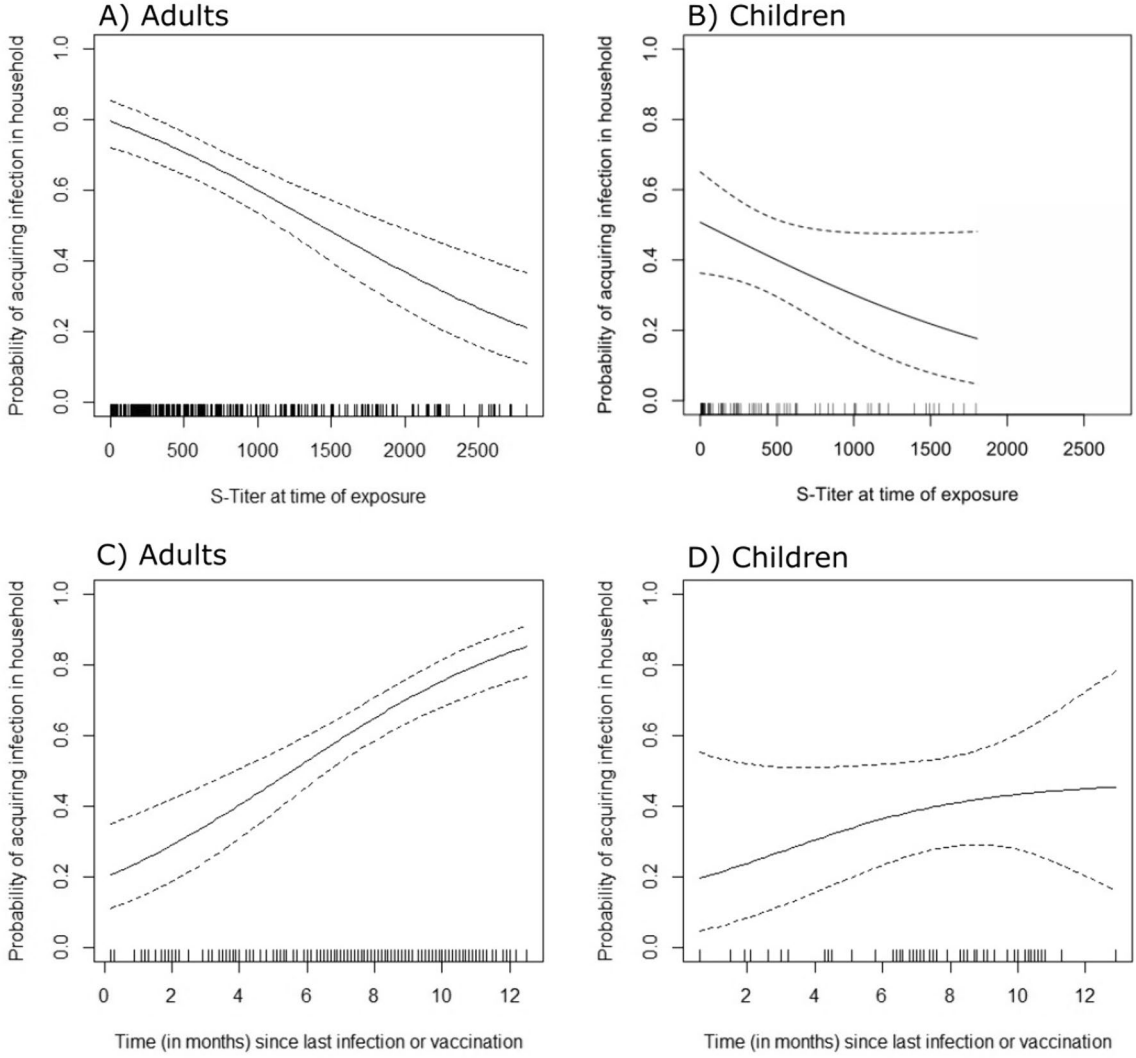
Probability of acquiring an infection after exposure in a household in relation to the protein S-titer at the time point of exposure in adults (**A**, n = 285) and children (**B**, n = 84) and by time since last exposure (vaccination or infection, **C** (n = 277), **D**(n = 71)), censored at upper 5% to avoid unstable estimation in area of sparse data

The risk of acquiring an infection from an index case was higher for those with a lower S-titer at time of exposure in the household. The association was approximately linear and similar for adults and children (Fig. 1A, B). Conversely, the risk of acquiring an infection increased similarly with the time since the last infection or vaccination (1C, D). Since we did not observe a difference between vaccinations/previous infections in the above analysis (Figures S2 and S3), we combined those.

### Probability of acquiring an infection after exposure in the household

In the univariable logistic regression, the odds of acquiring an infection increased with the time since the preceding vaccination or infection (OR = 1.18, 95% CI = 1.10; 1.26 per month) (Table 2). Similar effects were found when time since vaccination was studied in those for whom vaccination was the last exposure and when time since last infection was studied among those for whom previous infection was the last exposure (Table 2), therefore both were combined. Similarly, a lower S-titer at exposure was associated with higher odds of acquiring an infection. Prevention measures applied in the household and less severe symptoms of the index case reduced the risk of infection (Table 2). There was also a difference depending on household composition with lower transmission risk when exposed were children compared to adults.

**Table 2:** Probability of infection acquisition in a household transmission depending on different factors (univariable logistic regression—each variable is considered separately) from the perspective of exposed persons

Variable		N	Univariable odds ratio (95%CI)
Time since last vaccination or infection	Per 1 month	361	1.18 (1.10; 1.26)
For children		75	1.08 (0.95; 1.24)
For adults		286	1.22 (1.12; 1.33)
Time since last vaccination*	Per 1 month	213	1.12 (1.02; 1.24)
For children		27	1.05 (0.77; 1.43)
For adults		186	1.12 (1.01; 1.25)
Time since last infection**	Per 1 month	148	1.16 (1.04; 1.28)
For children		48	1.10 (0.95; 1.27)
For adults		100	1.24 (1.07; 1.45)
S-protein titer at time point of exposure	Per 100 BAU/ml increase	389	0.95 (0.93; 0.97)
Prevention measures in the household	Yes	228	Ref
	No	159	1.68 (1.11; 2.55)
Direction of transmission***	Adult to adult	239	Ref
	Child to adult	53	0.72 (0.39; 1.32)
	Adult to child	74	0.38 (0.22; 0.64)
	Child to child	13	0.34 (0.11; 1.09)
Index Case***			
	Adult	313	Ref
	Child	66	0.79 (0.46; 1.35)
Severity of symptoms of the case***	ARI	322	Ref
	Mild symptoms	62	0.74 (0.43; 1.28)
	No symptoms	5	0.46 (0.08; 2.81)

*Only those for whom vaccination was the last exposure before the exposure in the household to the index case

**Only those for whom infection was the last exposure before the exposure in the household to the index case

***From the perspective of those exposed (i.e. the 389 household members), for each exposed person multiple index cases were possible

In the multivariable analysis, the estimates were similar, indicating no substantial confounding among the studied factors (Table 3). Furthermore, the results of the sensitivity analysis classifying secondary cases with a 1.5-fold titer increase as not infected or additionally including cases with change from possible to positive titer showed similar estimates (Table S4).

**Table 3:** Association of time since last vaccination or infection / titer at exposure and other covariates with risk of acquiring infection (two multivariable logistic regression models—each model mutually adjusted for all considered variables)

Characteristics		Time since last infection or vaccination	S-titer at exposure
Time since last infection or vaccination (per month)		1.22 (1.13; 1.32)	–
Titer at exposure (per 100 units increase		–	0.94 (0.92; 0.96)
Severity of symptoms of the index case			
	ARI	Ref	
	Mild symptoms	0.78 (0.41; 1.51)	0.73 (0.39;1.35)
	No symptoms	0.41 (0.06; 2.66)	0.73 (0.06; 2.42)
Index case			
	Adult	Ref	
	Child	1.22 (0.64; 2.30)	1.16 (0.62; 2.15)
Sex			
	Male	Ref	
	Female	1.08 (0.68; 1.70)	0.90 (0.58; 1.40)
Age of exposed individual (per year)		1.02 (1.01; 1.03)	1.03 (1.02; 1.04)
Prevention measures			
	Yes	Ref	
	No	1.75 (1.09; 2.80)	1.66 (1.05; 2.61)

## Discussion

In this prospective study with exposure defined by an index case in the same household, we found that the probability of acquiring an infection in the household increased almost linearly with the time since last exposure from 20% shortly after a previous exposure to over 80% at one year after the last exposure (similarly for preceding infections and vaccinations). A similar linear relationship was observed for the declining antibody S-titer. Prevention measures in the household effectively mitigated the risk.

We showed that only short times after last exposures protected household members from infection. This is in line with a previous meta-analysis showing that protection against Omicron is substantially reduced only for a short time when the past infection was with a pre-Omicron variant [8]. Even if the past infection was with Omicron, the protection was low especially for BA.4 and BA.5 which was the predominant sublineage mid 2022 in Germany [8]. Similarly, to our findings, there was an almost linear decrease of protection during the first 12 months for Omicron (only shown for BA.1). A recent meta-analysis showed that protection solely through vaccination decreased very fast and hybrid immunity had the best protection [20]. Interestingly, a previous infection seemed to provide better protection than a vaccination. However, in our analysis the probability for acquiring infection was similar for participants with last exposures being infections or vaccinations.

One interesting aspect is the situation of children in household transmission. The probability of acquiring an infection increased with the time since the last exposure also for children but not as strong as for adults. However, since the number of children included in our study was low, these results had a higher uncertainty. Children also were less likely to be the index case in our sample and less likely to transmit or acquire infection compared to the transmission among adults. Lower transmission risk was particularly documented for children for the earlier variants [21, 22], but our data indicated this effect also for the Omicron, albeit not very strong.

We also found that adherence to prevention measures in households reduced transmission. This is in line with previous research and shows that it is possible to reduce risk of infections the household setting, e.g. one study found that the isolation of the index case was associated with a lower transmission risk [23]. At the same time, the relative effect appears considerable, but the absolute effect is not very strong. We did not focus on the topic in our study and asked only very broadly about prevention measures, so the findings should be treated with caution. Low standardization and small sample size precluded analyses of individual prevention measures.

The calculated SAR in our study was high compared to previous studies reporting SAR between 31-53% [4]. First, given that transmission depends on time since the last exposure, a different composition of the sample with respect to the last vaccination or infection will affect SAR. Second, studies with denser testing are likely to identify more mild symptomatic or asymptomatic cases compared to studies with less testing. This can be related to primary as well as secondary cases. As primary cases, we included only participants who reported a positive test. Thus our primary cases are more likely to have clear symptoms prompting testing. Since the risk of transmitting infection depended on symptoms of the index case, this could have inflated the estimates. On the other side, we could have missed some secondary cases with a weak immune response, as we did not conduct systematic testing during the potential transmission phase. However, we tried to overcome this limitation by including participants with a titer seroconversion or a strong titer increase as cases. With this approach, we might also have captured asymptomatic cases but also participants who were actually not infected but just showed a strong immune reaction when being exposed. Third, for simplification, we assumed that all secondary cases in a household resulted from the index case. In fact, there could have been some infection chains, i.e. the third person was not infected by the index case, but by a secondary case. Given this aspect, SAR and risk of transmission/acquisition of an infection in our study are the upper boundary. Still the linear form of the function should not be affected by this simplification.

We showed that a household transmission study can be conducted in a relatively short time even when incidence is low. We conducted the study completely remote and showed that self-sampling of blood is feasible under pandemic conditions. In contrast to most other household transmission studies, we used a prospective approach limiting the recall bias of participants and being able to measure changes in S-titer. Participants of DigiHero are from a community setting so that mostly mild cases are captured in this study. We did not determine the SARS-CoV-2 variant, however there was a strong dominance of Omicron during the study time [24].

Our study has some limitations. It is possible that we misclassified the cases e.g. the first mild symptomatic/asymptomatic case infected the “index” which then became symptoms and was tested positive. It might be also possible that household contacts had unknown SARS-CoV-2 exposures outside from the household and were misclassified as secondary cases from the household, although they have been independently infected. Given that only adults participate in DigiHero, we may have had more households where the index case was an adult, as they may have been more likely to think about participating in the transmission module if they had tested positive themselves. We also could have missed some cases, because we did not ask for systematic testing or a proof of positive tests. DBS have been proven as a valid alternative for blood collecting, however, in some cases, we received invalid results (79 participants). There are already some self-sampling systems to collect blood in tubes and this might improve the quality of serology results in the future. Additionally, we could not consider unknown previous infections of individuals, leading to a misclassification of the previous exposure of these cases.

In conclusion, the transmissibility of the omicron variant of SARS-CoV-2 in a household exposure is high. Vaccinations and preceding infections reduce the risk of transmission only for a relatively short period, supporting the notion of seasonal circulation of the virus. Fortunately, it appears from other sources that the protection against a severe course of the SARS-CoV-2 infection lasts longer than the protection against any infection.

## Supplementary Information

Below is the link to the electronic supplementary material.

Supplementary file1 (DOCX 345 KB)

## Data Availability

The anonymized data reported in this study can be obtained from the corresponding author upon request. The dataset includes individual data and an additional data dictionary will be provided. The beginning of data availability starts with the date of publication and the authors will support any requests in the three following years. Data requests should include a proposal for the planned analyses. Decisions will be made according to data use by the access committee of the DigiHero study, and data transfer will require a signed data access agreement.
