# Supplementary material for: Household transmission of Omicron variant of SARS-CoV-2 under conditions of hybrid immunity—a prospective study in Germany
Source: Infection. 2024 Jul 22;53(1):221–30. doi: 10.1007/s15010-024-02352-4 (PMC11825627; doi:10.1007/s15010-024-02352-4)
Supplement: Supplementary file 1 — Supplementary file1 (DOCX 345 KB) [file 15010_2024_2352_MOESM1_ESM.docx]

**Supplements**

**Tables**

**Table S1:** Number of household members with specific combinations of previous infections and vaccinations

|  | zero infections | one infection | two infections | Total |
| --- | --- | --- | --- | --- |
| zero vaccinations | 20 | 30 | 11 | 61 |
| one vaccination | 0 | 7 | 5 | 12 |
| two vaccinations | 18 | 39 | 5 | 62 |
| three vaccinations | 138 | 76 | 7 | 221 |
| four vaccinations | 21 | 9 | 0 | 30 |
| Missing |  |  |  | 3 |
| Total | 197 | 161 | 28 | 389 |

**Table S2:** Occurrence and severity of symptoms of secondary cases (n=224) by mode of detection

|  | | Total | No symptoms | Mild symptoms | ARI |
| --- | --- | --- | --- | --- | --- |
| Positive SARS-CoV-2 test | 142 | | 1 (0.7%) | 13 (9.2%) | 128 (90.1%) |
| Seroconversion | 24 | | 8 (33.3%) | 10 (41.7%) | 6 (25.0%) |
| 1.5 fold titre increase | 58 | | 18 (31.0%) | 26 (44.8%) | 13 (22.4%) |

**Table S3:** Use of prevention measures in the participating households

| Measures | Number of households (n=155) |
| --- | --- |
| Disinfection | 91 |
| Separate meal times | 83 |
| Staying in separate rooms | 124 |
| Wearing masks | 110 |
| Keep distance | 135 |
| Others | 13 |

**Table S4:** Sensitivity Analysis: Multivariable logistic regression excluding individuals with infection defined just on the basis of S-titer increase (Model 1) or defining them as not infected (Model 2)

| Model | Characteristics | Time since last infection or vaccination* | S-titer at exposure* |
| --- | --- | --- | --- |
| 1 | time since last infection or vaccination (per month) | 1.29 (1.17; 1.41) | - |
|  | Titer at exposure (per 100 units increase | - | 0.92 (0.89; 0.95) |
| 2 | time since last infection or vaccination (per month) | 1.26 (1.16; 1.37) | - |
|  | Titer at exposure (per 100 units increase | - | 0.93 (0.91; 0.96) |

*adjusted for severity of symptoms of the index case, index case, sex of exposed household contact, age of exposed household contact, and prevention measures

**Figures**


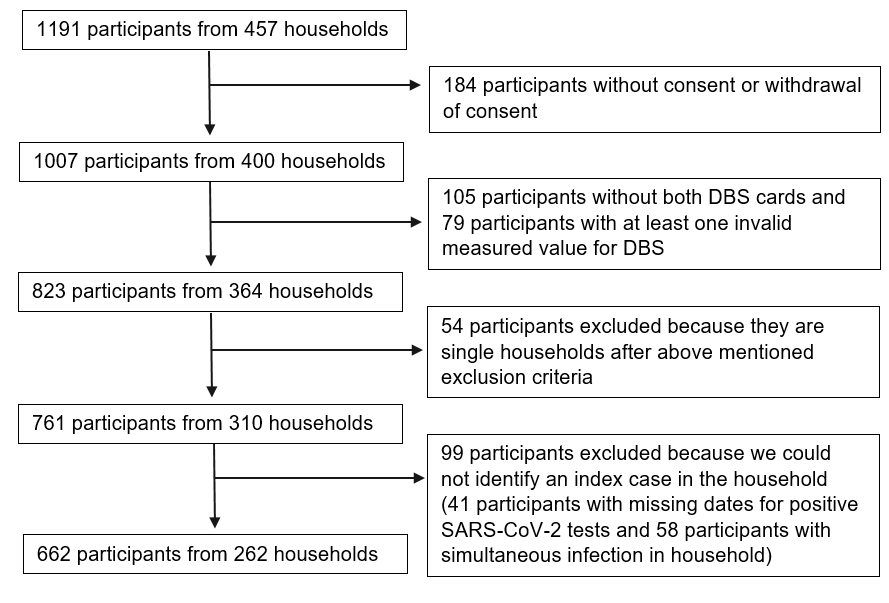


**Figure S1:** Flowchart for study participants in the transmission study


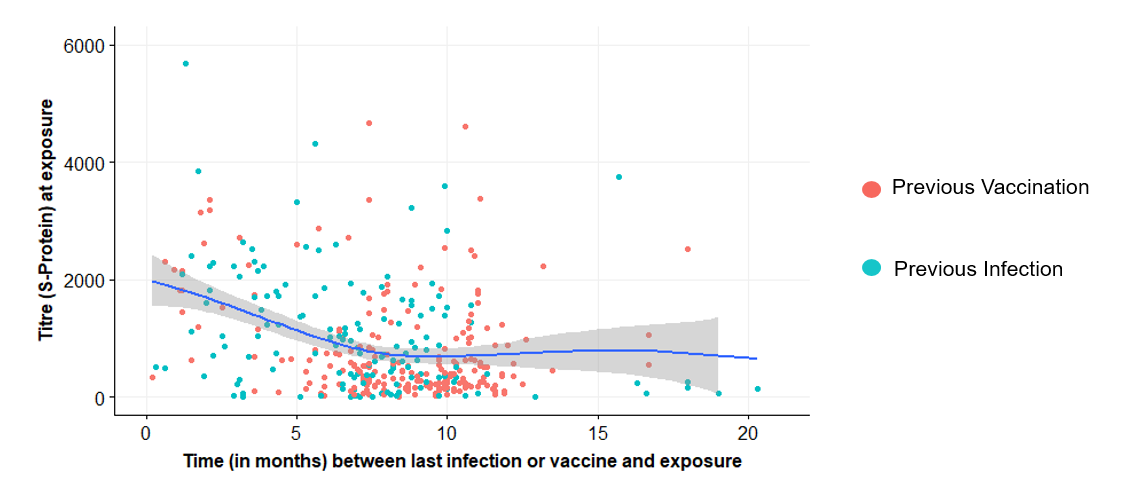


**Figure S2:** Distribution of titers and time since the last exposure (vaccination or previous infection) of all household members with previous exposure.


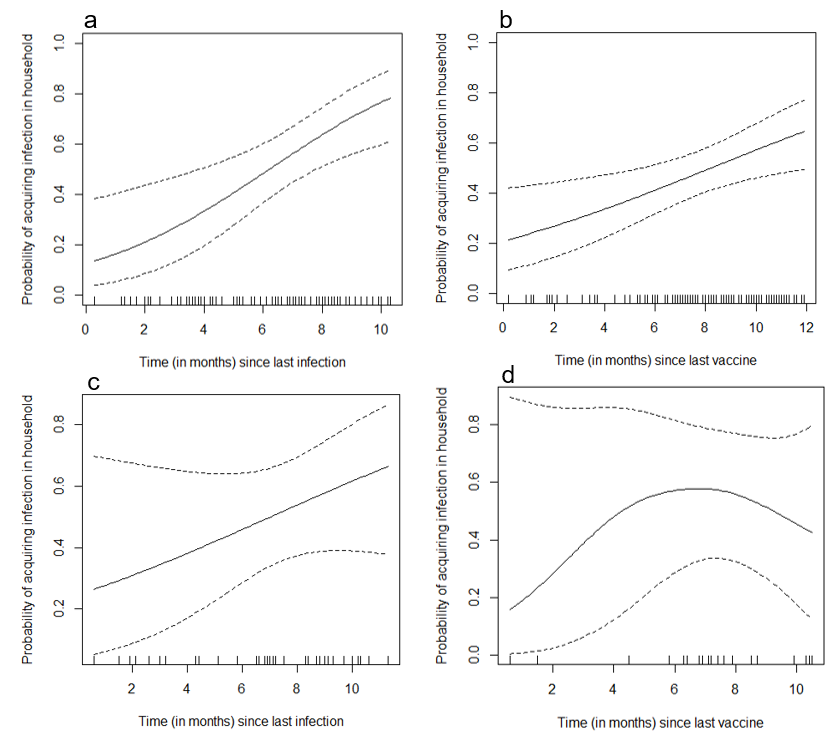


**Figure S3:** Time in months since last infection or vaccination for adults (a,b) and children (c,d), censored at upper 5% to avoid unstable estimation in the area of sparse data.
